# Fluctuations and interrelationship of oxidative stress and hepcidin during the menstrual cycle

**DOI:** 10.3389/fendo.2025.1689305

**Published:** 2025-10-22

**Authors:** Ena Yoshida, Harumi Hayashida, Tomomi Shimizu, Amanda J. Cox, Katsuhiko Suzuki

**Affiliations:** ^1^ Graduate School of Sport Sciences, Waseda University, Tokorozawa, Saitama, Japan; ^2^ Faculty of Sport Sciences, Toin University of Yokohama, Yokohama, Kanagawa, Japan; ^3^ Faculty of Biomedical Engineering, Toin University of Yokohama, Yokohama, Kanagawa, Japan; ^4^ Institute for Biomedicine and Glycomics, School of Pharmacy and Medical Sciences, Griffith University, Gold Coast, QLD, Australia; ^5^ Faculty of Sport Sciences, Waseda University, Tokorozawa, Saitama, Japan

**Keywords:** oxidative stress, menstrual cycle, iron metabolism, Hepcidin, iron deficiency, d-ROMs

## Abstract

**Background:**

Menstruation is a daily opportunity for iron loss in women. Hepcidin (Hepc), a key regulator of iron metabolism, is known to respond to both iron status and inflammation. Menstruation is also accompanied by local and systemic production of reactive oxygen species and inflammatory responses. However, fluctuations in Hepc and oxidative stress during the menstrual cycle and their relationship are unclear. The purpose of this study was to clarify of the fluctuations Hepc and oxidative stress and relationships.

**Methods:**

Sixteen women were recruited, of whom twelve with normal menstrual cycles were included in the final analysis. Blood samples were collected at four time points — The menstrual phase (MP), follicular phase (FP), early luteal phase (ELP), and late luteal phase (LLP) — while the participants were at rest. Serum Hepc, serum ferritin (FER), and oxidative stress levels were evaluated. In addition, differences between the iron-deficient (ID, <12 ng/ml) and non–iron-deficient (NID, ≥12 ng/ml) groups, classified according to FER levels during the ELP, were examined.

**Results:**

Oxidative stress showed significant fluctuations across the menstrual cycle (p < 0.01), with higher values during the MP and FP compared with the LLP. This trend was particularly pronounced in the ID group. Hepc did not exhibit significant cyclical fluctuations. Nevertheless, its mean level was highest in the MP and lowest in the FP. No significant correlation was observed between oxidative stress and Hepc. FER was positively correlated with Hepc only in the LP (r = 0.769, p = 0.043), and significant differences in Hepc levels between the ID and NID groups were observed exclusively in the ELP (p = 0.003) and LLP (p = 0.010).

**Conclusion:**

Oxidative stress fluctuated across the menstrual cycle, with increases observed during the MP and FP. These fluctuations appeared to be more pronounced in the presence of ID. In contrast, Hepc did not exhibit consistent cyclical changes. Although oxidative stress was considered to influence Hepc elevation through inflammatory responses, no direct relationship was detected at the blood marker level.

## 1 Introduction

The menstrual cycle is defined as the period from the start of menstruation to the start of the next menstruation. Women experience this cycle approximately every month from menarche at puberty until menopause (1). Menstrual bleeding is a common source of iron loss for women and affects iron metabolism (2, 3). Iron is an essential element involved in numerous biological functions, such as energy metabolism and enzyme reactions. However, iron deficiency, in which the body’s iron stores are depleted, is an extremely common health issue, affecting 15-30% of women (4–7). Iron deficiency profoundly affects women’s overall health and quality of life through chronic fatigue, decreased physical ability and concentration, and immune regulation disorders (8). Therefore, a deeper understanding of the mechanisms regulating recurrent iron loss during the menstrual cycle is essential for advancing women’s health.

Hepcidin (Hepc), a liver-derived peptide hormone, is a key regulator of systemic iron metabolism (9). Hepc is a hormone that inhibits iron storage in the body. When iron stores are depleted, Hepc secretion decreases, whereas sufficient iron stores stimulate its secretion (10, 11). In addition, Hepc is known to be strongly induced by inflammatory stimuli (12, 13). During menstruation, the endometrium undergoes detachment, hemostasis, and repair, and during this process, neutrophils and macrophages accumulate and infiltrate the tissue, generating large amounts of reactive oxygen species (ROS) (14–16). ROS activate NF-κB signaling pathways, thereby promoting the production of inflammatory cytokines. Thus, ROS act not only as oxidative stress factors but also as inducers of inflammatory responses (17–19). In fact, it has been reported that inflammatory cytokines in the blood increase in women during menstruation, and menstruation can be said to be an opportunity for systemic physiological inflammation through local ROS production (14, 16, 20–22). Excessive secretion of Hepc caused by inflammatory responses leads to excessive accumulation of iron in cells, which may also cause increased ROS production through the Fenton reaction in which ferrous iron catalyzes the conversion of hydrogen peroxide into highly reactive hydroxyl radicals (23, 24). Moderate ROS is essential for physiological phenomena, but disruption of the balance with its control system leads to oxidative stress (25). Impaired balance triggers cell damage and chronic inflammation (17, 26), contributing not only to the onset and progression of female-specific diseases, but also to further conditions such as cardiovascular disease (27–29).

Although several studies have individually observed fluctuations in oxidative stress or Hepc during the menstrual cycle, none have investigated them simultaneously or investigated their relationship. Therefore, the purpose of this study was to evaluate fluctuations in oxidative stress, assessed using the d-ROMs test that reflects the total amount of oxidative products generated by ROS, and Hepc dynamics associated with the menstrual cycle and to examine their relationship.

## 2 Materials and methods

### 2.1 Participants

No abnormalities were detected in the routine university health checkups, and healthy female university students without smoking habits or underlying diseases were recruited. Sixteen participants who had regular menstrual cycles and no history of oral contraceptive use volunteered to participate in this study. All participants received a detailed explanation of the study objectives and procedures, and written informed consent was obtained. This study was conducted in accordance with the Declaration of Helsinki and was approved by the Clinical Research Ethics Committee of Toin University of Yokohama (approval No. I-66).

Participants reported the onset of menstruation, with the first day designated as day 1. The menstrual phase (MP, days 1–3), follicular phase (FP, days 8–10), early luteal phase (ELP, days 15–17), and late luteal phase (LLP, days 22–24) were defined, and blood sampling and questionnaire surveys were conducted in each phase. The starting phase of data collection was randomized across participants, but all four measurements were completed within consecutive menstrual cycles. Twelve participants were included in the final analysis after excluding those whose cycle length was outside the range of 25–38 days based on one cycle. None of the participants took antioxidant or iron supplements during the study.

### 2.2 Physical activity survey

Starting from the commencement of measurement, participants were instructed to wear a physical activity monitor (LifeCoder EX, Suzuken, Japan) on their waist for one week and to record their daily physical activity (step counts). The monitor was worn during all daily activities except bathing and sleeping. The LifeCoder EX detects body vibrations and automatically records daily step counts, which can be stored for several consecutive days.

### 2.3 Collection and analysis of blood samples

Blood samples were drawn from antecubital region by a clinical laboratory technician. Participants were instructed to eat a meal 4 h before each sampling and to ensure that the same meal was consumed on all four occasions.

Samples were collected in heparinized, as instructed by the outsourcing company for the measurement of oxidative stress, and serum separator tube. Plasma and serum were separated by centrifugation and stored at −80°C until analysis.

Analyses of blood samples were outsourced as follows. Diacron reactive oxygen metabolites (d-ROMs), an oxidative stress marker, to Wismer Co., Ltd. The d-ROMs test is a method that primarily measures organic hydroperoxides present in serum or plasma using a colorimetric assay, which are early oxidation products generated by the oxidation of lipids, proteins, and nucleic acids. The obtained values reflect the degree of oxidative damage. Results are expressed in arbitrary units called U.CARR, where 1 U.CARR corresponds to the chemical equivalent of 0.08 mg/100 ml hydrogen peroxide (30). Serum ferritin (FER), a marker of iron stores, 17β-estradiol (E_2_) and pregnen-4-ene-3,20-dione (P_4_), to SRL, Inc. FER was measured using chemiluminescent enzyme immunoassay, with a reference range for women of 3.6–340 ng/ml, as provided by SRL, Inc. E2 and P4 were measured using electrochemiluminescence immunoassay; according to SRL, Inc., reference ranges for non-pregnant women are 28.8–196.8 pg/ml in the follicular phase, 36.4–525.9 pg/ml in the peri-ovulatory phase, and 44.1–491.9 pg/ml in the luteal phase for E2, and ≤0.28 ng/mL in the follicular phase, ≤5.69 ng/ml in the peri-ovulatory phase, and 2.05–24.2 ng/ml in the luteal phase for P4. Hepc, a hormone regulating iron homeostasis, was measured by Nikken Seil Co., Ltd. using the Quantikine Human Hepcidin ELISA (R&D Systems) on a Tecan Infinite M200 microplate reader.

### 2.4 Survey on menstrual symptoms

Menstrual symptoms were assessed in each phase using the Menstrual distress questionnaire (MDQ) (31, 32). The MDQ consists of 47 items addressing physical and psychological changes associated with the menstrual cycle. In each phase, participants rated their symptoms on a 4-point scale (0 = none, 1 = mild, 2 = moderate, 3 = severe). The total score, ranging from 0 to 141, was used as an index of symptom severity.

### 2.5 Subgroup analysis based on iron status

From the blood analysis, it was found that the largest number of participants with FER levels below the clinical criterion for iron deficiency (12 ng/ml) appeared in the ELP (33, 34). Accordingly, participants were divided into an iron-deficiency (ID) group (FER < 12 ng/ml; n = 5) and a non–iron-deficiency (NID) group (FER ≥ 12 ng/ml; n = 7), and exploratory analyses were performed.

### 2.6 Statistics analysis

Data are presented as mean ± standard deviation (SD) unless otherwise specified. The normality of each variable was assessed using the Kolmogorov–Smirnov test.

FER, E_2_, d-ROMs, Hepc, and MDQ scores satisfied normality and were analyzed using one-way analysis of variance (ANOVA) to examine fluctuations across the four time points. Two-way repeated-measures ANOVA was additionally performed for E_2_, d-ROMs, and MDQ scores to evaluate subgroup variations. Physical activity, which also satisfied normality, was compared between groups using independent samples t-tests.

In contrast, P_4_ did not meet the assumption of normality and was analyzed using the Friedman test for comparisons across the four time points and the Mann–Whitney U test for between-group comparisons. Similarly, subgroup analyses of Hepc did not satisfy normality. Therefore, the Friedman test and Mann–Whitney U test were applied.

Associations between variables (FER and Hepc, d-ROMs and Hepc) were assessed using Pearson’s correlation coefficient. When significant differences were identified by ANOVA or the Friedman test, *post hoc* analyses were performed with the Bonferroni correction.

For the Mann–Whitney U test, the corrected significance threshold was set at p < 0.0125, whereas for all other analyses statistical significance was defined as p < 0.05. All analyses were conducted using SPSS Statistics, version 30.0.0.0 (IBM, Ltd.).

## 3 Results

### 3.1 Participants characteristics

#### 3.1.1 Basic information about participants

The mean age of the participants was 19.8 ± 1.0 years with recorded body mass during the measurement period 52.6 ± 4.0 kg. The menstrual cycle of the participants was 29.8 ± 3.3 days, and measurements were taken on day 2.3 ± 0.9 of the MP, day 9.1 ± 0.9 of the FP, day 16.1 ± 0.9 of the ELP, and day 22.8 ± 0.9 of the LLP. The participants’ daily physical activity was 11,607.1 ± 3,631.0 steps, and no significant difference was observed between the ID group and the NID group (p = 0.783).

#### 3.1.2 Changes in E_2_ and P_4_

**Figure 1:**
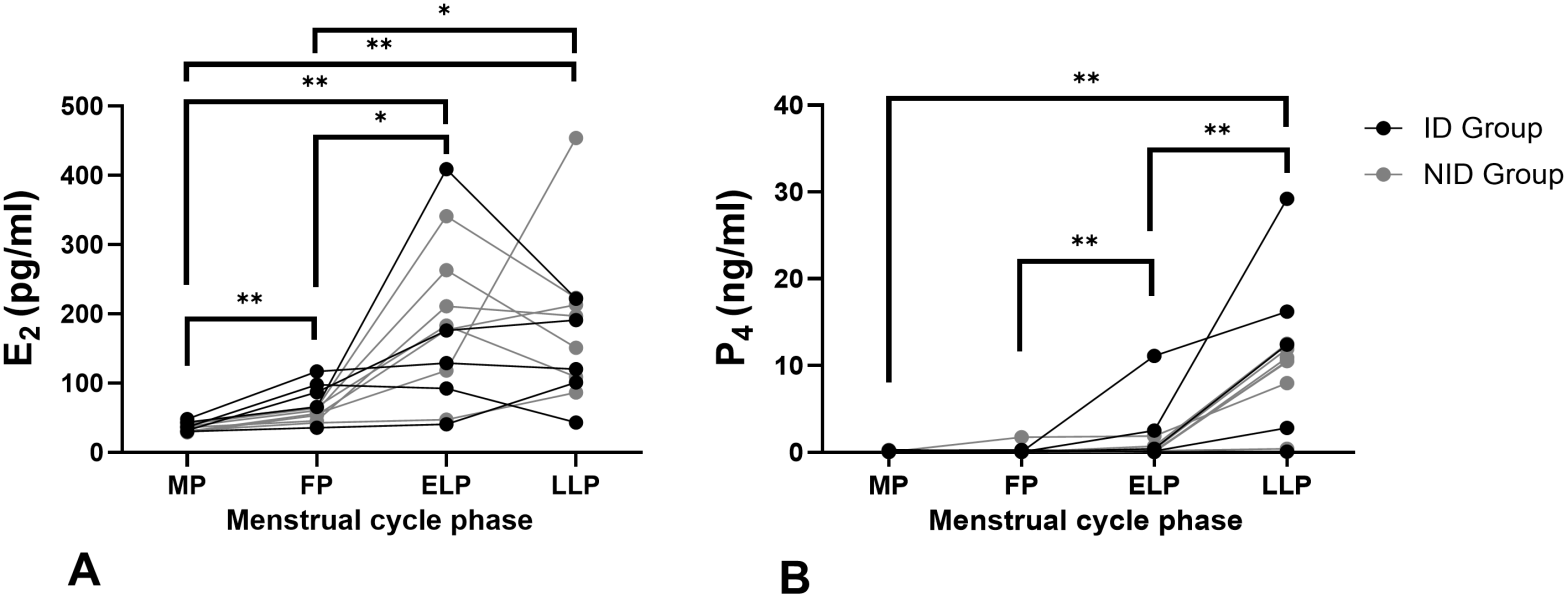
Changes in E_2_ and P_4_ during the menstrual cycle. **(A)** Changes in E_2_ concentrations and **(B)** changes in P_4_ concentrations. The asterisks indicate the results of one-way ANOVA with *post hoc* tests (*p < 0.05, **p < 0.001). Each line represents individual values (n = 12). Black indicating the iron-deficiency (ID) group and gray indicating the non–iron-deficiency (NID) group. E_2_: 17β-estradiol; P_4_: Pregn-4-ene-3,20-dione. MP, Menstrual phase; FP, Follicular phase; ELP, Early luteal phase; LLP, Late luteal phase.

**Figure 1** shows the changes in E_2_ and P_4_ concentrations across the menstrual cycle. The mean E_2_ concentrations were 36.54 ± 6.28 pg/ml in MP, 66.01 ± 23.74 pg/ml in LP, 182.27 ± 111.74 pg/ml in ELP, and 175.89 ± 105.81 pg/ml in LLP, showing significant variation across phases (p < 0.001). The mean P_4_ concentrations were 0.15 ± 0.10 ng/ml in MP, 0.21 ± 0.46 ng/ml in LP, 1.47 ± 3.00 ng/ml in ELP, and 10.44 ± 7.46 ng/ml in LLP, also demonstrating significant variation across phases (p < 0.001). Although the luteal phase increase in P4 was not clearly evident in some participants, overall significant changes were observed across the cycle. No significant differences in E_2_ or P_4_ were observed between the ID and NID groups in any phase.

### 3.2 Iron stores

**Figure 2:**
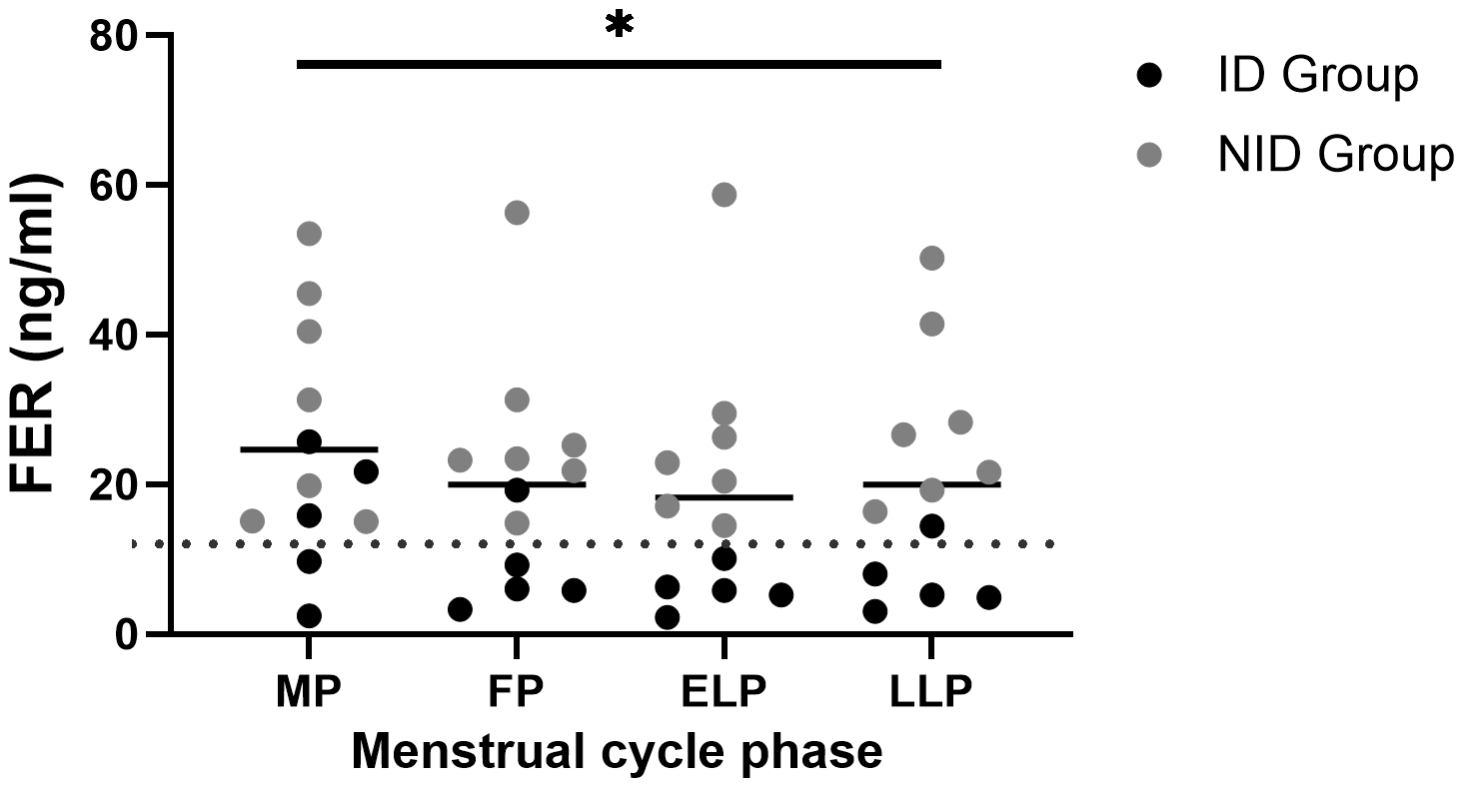
Fluctuations in FER during the menstrual cycle. The asterisk indicates the results of one-way ANOVA (*p < 0.05). Black dots indicate the iron-deficiency (ID) group, and gray dots indicate the non–iron-deficiency (NID) group. Horizontal bars indicate mean values (n = 12). MP, Menstrual phase; FP, Follicular phase; ELP, Early luteal phase; LLP, Late luteal phase; FER, Serum ferritin.

FER levels were 24.7 ± 14.6 ng/ml in the MP, 20.0 ± 13.9 ng/ml in the FP, 18.3 ± 14.9 ng/ml in the ELP, and 19.9 ± 14.2 ng/ml in the LLP, showing significant variation across the menstrual cycle (p = 0.049), with the highest mean value in the MP and the lowest in the ELP (**Figure 2**). However, no significant differences were detected in *post hoc* comparisons.

### 3.3 Oxidative stress

**Figure 3:**
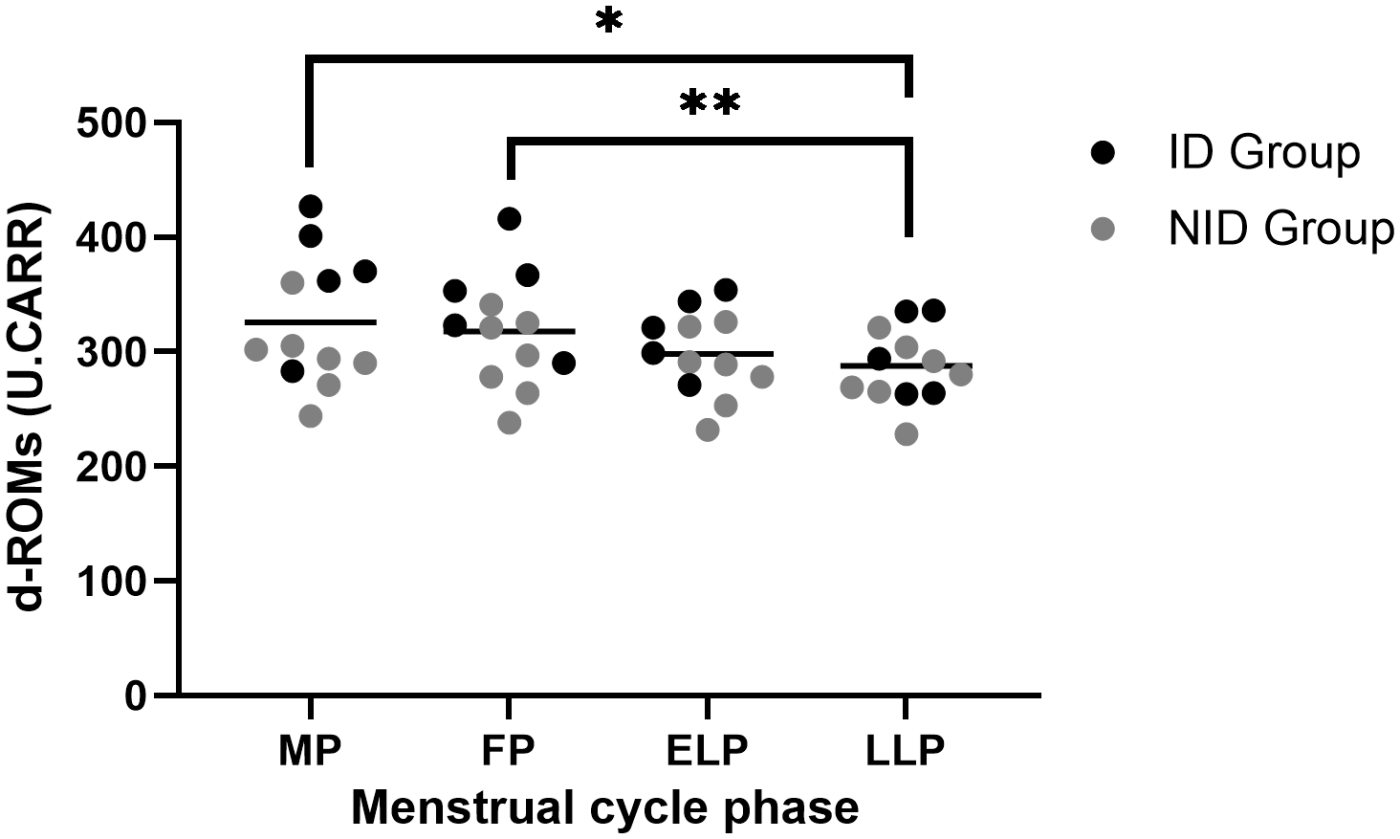
Fluctuations in d-ROMs during the menstrual cycle. The asterisks indicate the results of one-way ANOVA *post hoc* tests (*p < 0.05, **p < 0.01). Black dots represent the iron-deficiency (ID) group, and gray dots represent the non–iron-deficiency (NID) group. Horizontal bars indicate mean values (n = 12). 1 U.CARR corresponds to 0.08 mg/100 ml hydrogen peroxide equivalent. MP, Menstrual phase; FP, Follicular phase; ELP, Early luteal phase; LLP, Late luteal phase.

The d-ROMs showed significant fluctuations across the menstrual cycle (p < 0.01). *Post hoc* comparisons revealed significant differences between the MP and LLP (p = 0.046) and between the FP and LLP (p = 0.01) (**Figure 3**).

Exploratory subgroup analyses using two-way repeated-measures ANOVA revealed a significant group × menstrual cycle interaction (p = 0.04). *Post hoc* comparisons revealed that the ID group had significantly higher values than the NID group in the MP (p = 0.017). Similarly, in the FP, the ID group showed significantly higher values than the NID group (p = 0.047).

### 3.4 Iron regulation

**Figure 4:**
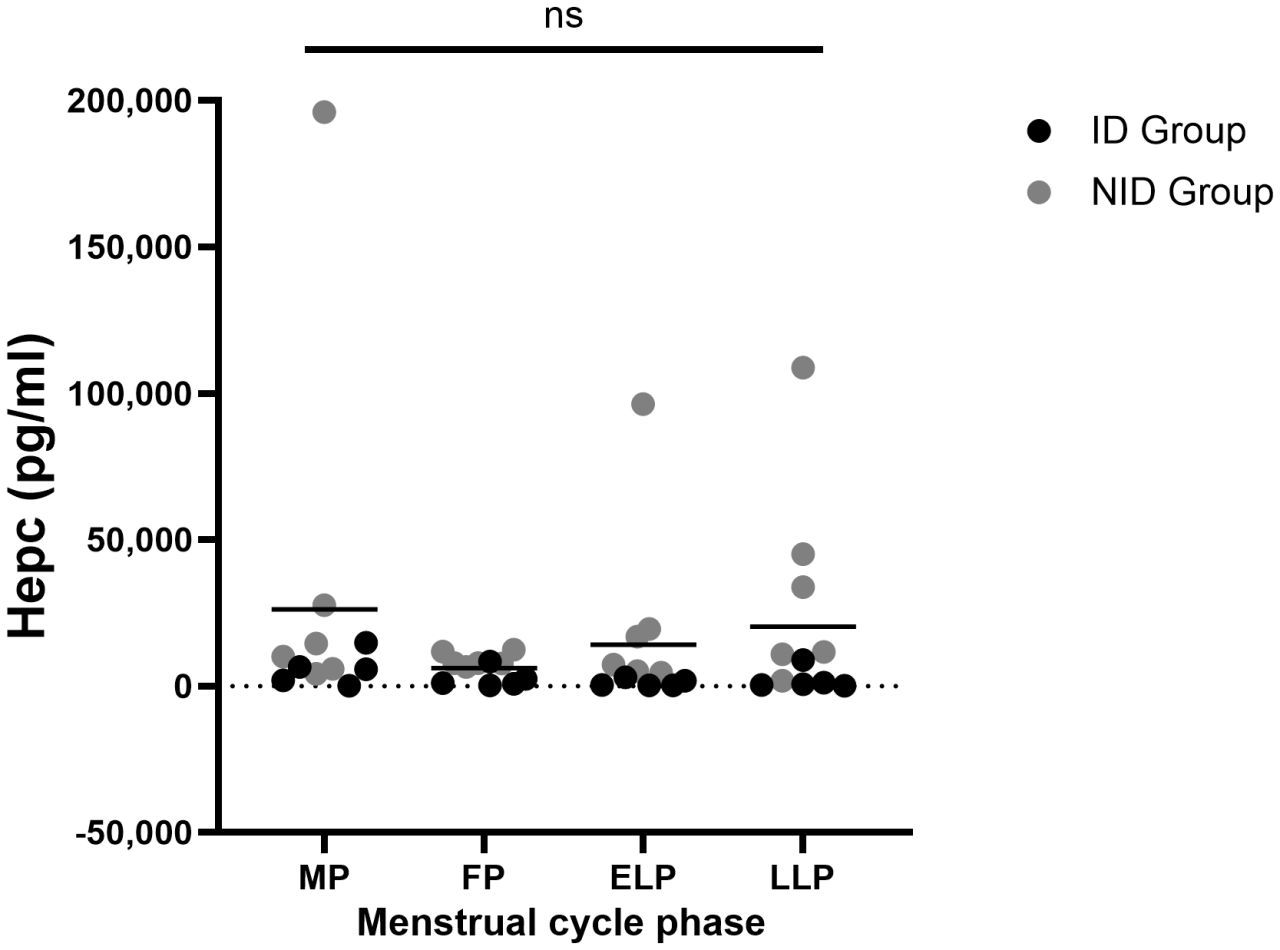
Fluctuations in Hepc during the menstrual cycle. ns indicates not significance in one-way ANOVA. Black dots represent the iron-deficiency (ID) group, and gray dots represent the non–iron-deficiency (NID) group. Horizontal bars indicate mean values (n = 12). MP, Menstrual phase; FP, Follicular phase; ELP, Early luteal phase; LLP, Late luteal phase; Hepc, Hepcidin.

Hepc did not fluctuate significantly across the menstrual cycle (p = 0.483) (**Figure 4**). The mean value was highest in the MP at 26,286.2 ± 54,214.0 pg/ml, and lowest in the FP at 6,096.6 ± 4,135.0 pg/ml. The mean values in the ELP and LLP were 20,634.1 ± 34,980.8 pg/ml and 19,109.4 ± 31,609.5 pg/ml, respectively.

In the subgroup analysis of Hepc, no significant fluctuations were observed across the four time points within each group (ID group: p = 0.178; NID group: p = 0.615). However, significant between-group differences were found in the ELP (p = 0.003) and the LLP (p = 0.010) (**Table 1**).

**Table 1:** Median Hepc and difference between ID and NID groups at each cycle.

	ID Group (n= 5)		NID Group (n=7)		P - value	Significance
MP	2,257.1	(1,988 – 6,585.4)	14,563.7	(7,979.1 – 27,815.8)	0.106	ns
FP	991.9	(817.2 – 2,502.6)	7,785.6	(7,129.1 – 9,816.0)	0.048	ns
ELP	332.7	(275.4 – 1,837.8)	16,920.3	(6,200.6 – 55,890.7)	0.003	*
LLP	644.6	(443.0 – 1,156.9)	11,590.7	(8,439.7 – 39,467.9)	0.010	*

Units are expressed in pg/ml. Data are presented as median (min–max). Mann–Whitney U test was used, with significance set at p < 0.0125. ns, not significant; *p < 0.0125. MP, Menstrual phase, FP, Follicular phase; ELP, Early luteal phase; LLP, Late luteal phase; ID, iron-deficiency; NID, non–iron-deficiency.

### 3.5 Correlations between FER and Hepc, and between d-ROMs and Hepc

**Table 2** shows the correlations between FER and Hepc, as well as between d-ROMs and Hepc, in each phase. A significant positive correlation between FER and Hepc was observed in the ELP (r = 0.769, p = 0.043), but not in the other phases. No significant correlations were observed between d-ROMs and Hepc in any phase (**Table 2**).

**Table 2:** Correlation between FER and Hepc, and between d-ROMs and Hepc in each phase.

	FER vs Hepc		d-ROMs vs Hepc	
	r	P - value	r	P - value
MP	0.308	0.501	-0.194	0.545
FP	-0.362	0.424	-0.247	0.440
ELP	0.769	0.043*	0.060	0.854
LLP	-0.164	0.725	0.590	0.856

r, correlation coefficient; *p < 0.05. Correlations were examined between values within each phase (n = 12). Relationships across phases were not assessed. MP, Menstrual phase; FP, Follicular phase; ELP, Early luteal phase; LLP, Late luteal phase; FER, Serum ferritin. Hepc: Hepcidin.

### 3.6 Menstrual symptom

**Figure 5:**
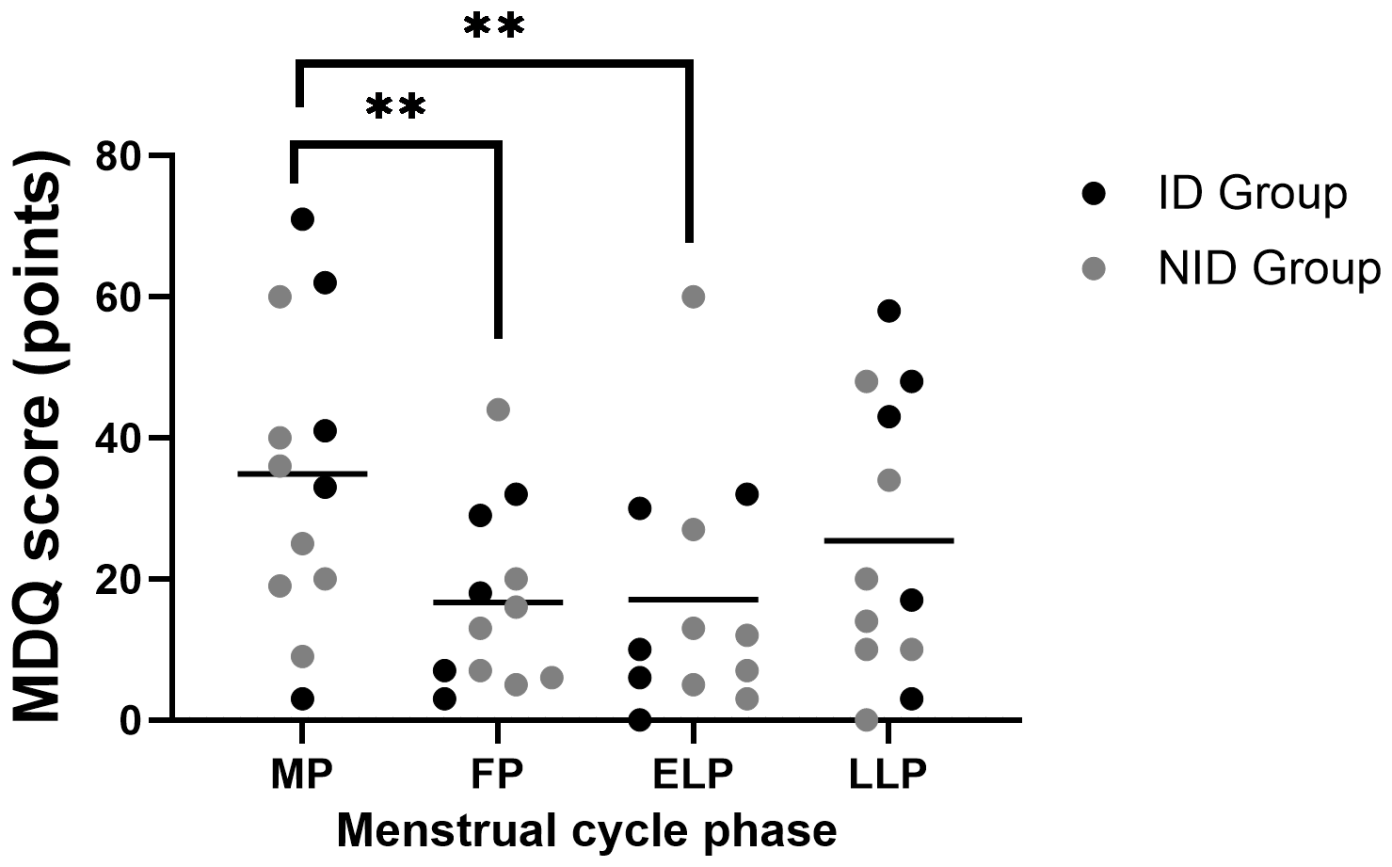
Fluctuations in MDQ scores during the menstrual cycle. Asterisks indicate the results of one-way ANOVA *post hoc* tests (**p < 0.01). Black dots represent the iron-deficiency (ID) group, and gray dots represent the non–iron-deficiency (NID) group. Horizontal bars indicate mean values (n = 12). MP, Menstrual phase; FP, Follicular phase; ELP, Early luteal phase; LLP, Late luteal phase.

MDQ scores showed significant fluctuations across the menstrual cycle (p < 0.001). *Post hoc* comparisons indicated significantly higher scores in the MP compared with the FP (p = 0.003) and the ELP (p = 0.004) (**Figure 5**).

Exploratory subgroup analyses using two-way repeated-measures ANOVA, group × menstrual cycle interaction showed a trend toward significance (p = 0.071).

## 4 Discussion

In this study, we simultaneously evaluated oxidative stress and Hepc dynamics during the menstrual cycle and examined their relationship. The results showed that oxidative stress was higher during the MP and FP compared to the LLP (**Figure 3**), and this was particularly pronounced in the ID group. Hepc did not show significant cyclical fluctuations (**Figure 4**), but the average values were highest during the MP and lowest during the FP. Although no direct correlation was observed between oxidative stress and Hepc (**Table 2**), the findings of this study indicate that oxidative stress and iron metabolism may be affected by the physiological fluctuations inherent to the female menstrual cycle.

Previous studies have reported inconsistent results regarding fluctuations in oxidative stress across the menstrual cycle (35–37), likely reflecting differences in the choice of oxidative stress markers and in the criteria used to define menstrual cycle, which complicate direct comparisons. In the present study, we found that oxidative stress was significantly higher in the MP and FP compared with the LLP (**Figure 3**). Several physiological mechanisms may underlie this pattern, which we discuss below.

First, menstruation is characterized by the infiltration of inflammatory cells, including neutrophils and macrophages, into the endometrium, accompanied by the activation of inflammatory and tissue repair processes (14–16). These cells generate ROS during this phase, which may contribute to systemic changes detectable in circulating oxidative stress markers. Second, the MP and FP are characterized by low levels of estrogen and progesterone, whereas their secretion increases during the ELP and LLP. Estrogen exerts antioxidant effects not only through the direct scavenging of ROS based on its phenolic structure but also by enhancing intracellular signaling pathways and the expression of antioxidant enzymes (38–40). Progesterone has similarly been reported to exert antioxidant effects (38). Therefore, during the ELP and LLP, the antioxidant effects of these hormones may have suppressed oxidative stress, which could partly explain why oxidative stress was more pronounced in the MP and FP compared with the luteal phases (**Figure 3**). Third, in the ID group, d-ROMs levels during the MP and FP were significantly higher than in the NID group, suggesting that iron deficiency may exacerbate the increase in oxidative stress observed during menstruation. Catalase, a key antioxidant enzyme that plays a central role in the defense against oxidative stress, contains iron at its active site. Indeed, reduced catalase activity and increased oxidative stress have been reported in patients with iron deficiency (41), and studies in iron-depleted cells and mouse models have also demonstrated increased oxidative damage in tissues (42, 43). Taken together, these findings suggest that in iron-deficient women, the reduced capacity to eliminate ROS during the MP and FP may have contributed to the greater increase in oxidative stress observed during these phases.

Hepc did not show significant cyclic fluctuations, although the mean values tended to be highest during the MP and lowest during the FP (**Figure 4**). Previous studies that reported significant cyclic variation in Hepc have shown a transient decrease after menstruation in response to iron loss (44, 45). In contrast, other studies have found no clear cyclic changes (46–48). Hepc secretion is regulated by both the BMP/SMAD pathway, which senses hepatic iron stores, and the IL-6/STAT3 pathway, which responds to inflammatory stimuli (49, 50) Given the considerable inter-individual differences in menstrual blood loss and inflammatory responses during menstruation (51, 52), these factors may have complicated the Hepc response and obscured consistent fluctuations in our study.

Regarding FER fluctuation during menstrual cycle, previous studies have reported inconsistent findings, with some showing the lowest levels during menstruation and others indicating rather elevated levels (46, 53–55). In this study, there was a statistically significant overall effect of the menstrual cycle on FER (**Figure 2**). However, *post hoc* tests did not reveal clear phase-specific differences. The mean values were highest during the MP and lowest during the ELP. FER is not only an indicator of iron stores but also an acute-phase protein that increases in response to inflammation (56, 57). ROS are known to act as inducers of inflammatory responses through the activation of NF-κB pathways (15, 17, 18, 58–60). In this study, the elevated FER observed during the MP was accompanied by increased oxidative stress, suggesting that it reflected not merely an increase in iron storage but rather an inflammatory response. This inflammatory response may, in turn, have promoted Hepc secretion and contributed to the higher Hepc levels observed during menstruation (**Figure 4**).

Additionally, significant differences in Hepc levels were observed between the ID group and the NID group during the ELP and the LLP (**Table 1**). In the ID group, despite being in an iron-deficient state during the MP and subsequent FP, inflammation may have increased FER and Hepc levels, thereby obscuring the differences in Hepc values compared with the NID group. Furthermore, a significant correlation between FER and Hepc was observed only in the ELP (**Table 2**). Given that estrogen concentrations, which have antioxidant and anti-inflammatory effects, were highest during this period (**Figure 1**) (38–40), it is thought that Hepc induction by inflammatory cytokines weakened, making it easier to reflect iron storage status.

Because oxidative stress can contribute to Hepc production through inflammatory pathways, we expected to observe a relationship. However, d-ROMs did not show significant correlations with Hepc in any phase (**Table 2**). Oxidative stress itself is not a direct regulator of Hepc (17, 18), and its effects are likely to be indirect and mediated. Moreover, the d-ROMs assay used in this study responds rapidly to acute changes in oxidative stress and tends to return to baseline within several tens of minutes to a few hours (61, 62). In contrast, Hepc is induced by inflammatory stimuli such as IL-6 over a period of several hours up to 24 hours (63, 64). The characteristics of this oxidative stress marker and the temporal mismatch in the Hepc response may have prevented us from detecting a relationship in this single-sample collection study. Therefore, to clarify the hypothesis that oxidative stress contributes to Hepc induction, observation through serial sampling over time will be necessary.

The MDQ score was highest during the MP, and it tended to be higher in the ID group than in the NID group (**Figure 5**). This suggests that oxidative stress, inflammatory responses, and iron deficiency may be involved in the exacerbation of menstrual-related symptoms, and further investigation of this relationship is warranted.

This study is novel in that it simultaneously evaluated systemic oxidative stress and Hepc fluctuations during the menstrual cycle, which had previously been examined separately, and clarified the relationship between their dynamics and iron metabolism. These findings provide fundamental insights that may contribute to women’s health management, particularly when combined with more comprehensive and longitudinal evaluations in future studies. Furthermore, future investigations examining how targeted interventions, such as antioxidant supplementation or iron fortification during specific menstrual phases, influence iron metabolism could help establish more effective iron supplementation strategies and personalized interventions for women at high risk of iron loss, such as those with heavy menstrual bleeding or athletes with high iron turnover.

Nevertheless, this study had a small sample size, and subgroup analysis was positioned as an exploratory observational study. Additionally, there are certain limitations in determining the menstrual cycle, as long-term monitoring and ovulation confirmation were not performed, and strict exclusion based on hormonal levels was not applied (65, 66). However, by conducting four repeated measurements within a single cycle for the same subjects, reliability was ensured to the greatest extent possible. Future studies with more rigorous menstrual cycle evaluation will help ensure reproducibility and strengthen the generalizability of these findings.

## 5 Conclusions

In this study, we evaluated oxidative stress and Hepc dynamics across menstrual cycle and their relationship. Oxidative stress increased during the MP and FP, and was further exacerbated by iron deficiency. In contrast, Hepc showed no clear cyclical variation, in significant, possibly due to individual differences in oxidative stress, inflammatory responses, and menstrual blood volume. During the ELP, Hepc a positive correlation between Hepc and FER, and group differences between the ID and NID were observed only in the ELP and LLP, suggesting cycle-specific effects. Although oxidative stress was expected to elevate Hepc via inflammation, this was not clearly demonstrated in the blood markers.

## Data Availability

The raw data supporting the conclusions of this article will be made available by the authors, without undue reservation.
